# Automated tissue Doppler imaging for identification of occluded coronary artery in patients with suspected non-ST-elevation myocardial infarction

**DOI:** 10.1007/s10554-022-02786-7

**Published:** 2023-01-30

**Authors:** Marlene Iversen Halvorsrød, Gabriel Kiss, Thomas Dahlslett, Asbjørn Støylen, Bjørnar Grenne

**Affiliations:** 1 Center for Innovative Ultrasound Solutions, Department of Circulation and Medical Imaging (ISB), Faculty of Medicine and Health Sciences (MH), Norwegian University of Science and Technology (NTNU), Trondheim, Norway; 2 Clinic of Cardiology, St. Olavs University hospital, Trondheim, Norway; 3 Department of Computer Science (IDI), Faculty of Information Technology and Electrical Engineering (IE), Norwegian University of Science and Technology (NTNU), Trondheim, Norway; 4 Sørlandet hospital, Arendal, Norway

**Keywords:** Post systolic shortening, Strain, Strain rate, Ischemic heart disease, Speckle tracking, Tissue Doppler imaging

## Abstract

**Purpose:**

Identification of regional dysfunction is important for early risk stratification in patients with suspected non-ST-elevation myocardial infarction (NSTEMI). Strain echocardiography enables quantification of segmental myocardial deformation. However, the clinical use is hampered by time-consuming manual measurements. We aimed to evaluate whether an in-house developed software for automated analysis of segmental myocardial deformation based on tissue Doppler imaging (TDI) could predict coronary occlusion in patients with suspected NSTEMI.

**Methods:**

Eighty-four patients with suspected NSTEMI were included in the analysis. Echocardiography was performed at admission. Strain, strain rate and post-systolic shortening index (PSI) were analyzed by the automated TDI-based tool and the ability to predict coronary occlusion was assessed. For comparison, strain measurements were performed both by manual TDI-based analyses and by semi-automatic speckle tracking echocardiography (STE). All patients underwent coronary angiography.

**Results:**

Seventeen patients had an acute coronary occlusion. Global strain and PSI by STE were able to differentiate occluded from non-occluded culprit lesions (respectively − 15.0% vs. -17.1%, and 8.1% vs. 5.1%, both p-values < 0.05) and identify patients with an acute coronary occlusion (AUC 0.66 for both strain and PSI). Measurements of strain, strain rate and PSI based on TDI were not significantly different between occluded and non-occluded territories.

**Conclusion:**

Automated measurements of myocardial deformation based on TDI were not able to identify acute coronary occlusion in patients with suspected NSTEMI. However, this study confirms the potential of strain by STE for early risk stratification in patients with chest pain.

## Introduction

Identification of regional myocardial dysfunction is important for the early risk stratification in patients with suspected non-ST-elevation myocardial infarction (NSTEMI). Strain echocardiography enables quantification of regional deformation and has evolved as a promising tool to identify patients with suspected NSTEMI who have an occluded culprit coronary artery [1–6]. Echocardiography is widely available and provides real-time imaging of cardiac function. However, current methods for strain measurements are time-consuming and operator dependent. Moreover, different techniques exist for myocardial deformation measurements.

Strain is mostly analyzed by speckle tracking echocardiography (STE), based on speckle recognition and motion tracking from frame to frame in B-mode images. Modern software allows semi-automatic measurements of STE. Strain can also be calculated by tissue Doppler imaging (TDI), which has the important advantage of higher frame rates [7]. High frame rates make it possible to detect rapid changes in deformation, which is important for correct measurement of peak strain, post systolic shortening (PSS), and strain rate. Furthermore, high frame rate is important for deformation tracking in patients with rapid heart rates. Nevertheless, TDI suffers from noise and measurements are dependent on beam direction. In contrast to strain by STE, strain by TDI is based on manual, time-consuming interpretation of deformation curves, which also increases operator variability in post processing. Automatization could make TDI more clinically useful by reducing post processing time and reducing operator variability.

A new software capable of extracting tissue velocities from TDI in the apical views, interpolate the velocities into 3D and automatically extract strain and strain rate has been developed [8]. The aim of this study was to evaluate the ability of the new automated TDI software to predict coronary occlusion in patients with suspected NSTEMI and compare the results to manual TDI-based measurements and commercially available STE analyses.

## Methods

### Study design

**Fig. 1:**
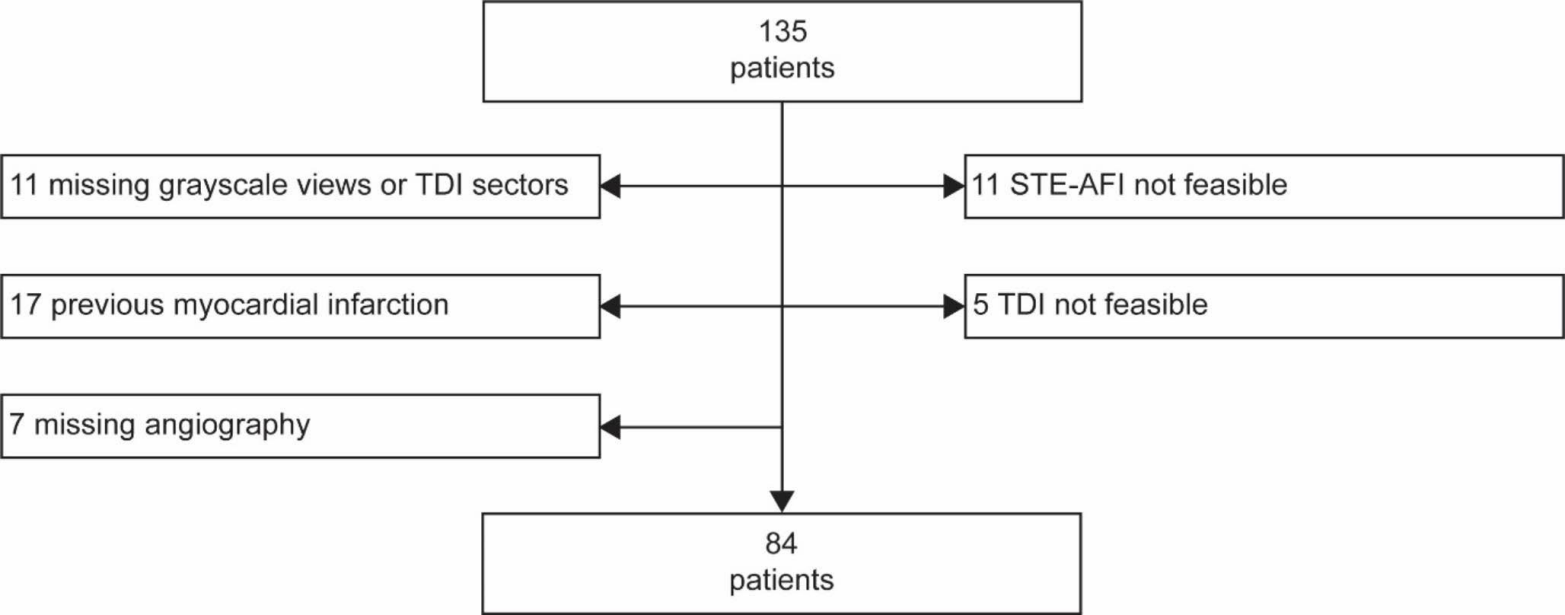
Flow diagram of the study population. TDI = tissue Doppler imaging. STE-AFI = speckle tracking echocardiography Automated Functional Imaging

One hundred thirty-five eligible patients referred to a hospital with suspected NSTEMI with acute anginal pain lasting for ≥ 10 min, a history of < 3 days and indication for coronary angiography according to guidelines [9] were screened. Patients with previous MI (n = 17), missing angiography (n = 7), missing greyscale views or TDI sectors (n = 11) were excluded (Fig. 1). The final study population was 100. All included patients underwent echocardiographic examination and coronary angiography. The study was approved by the Regional Committee for Medical Research Ethics and conducted according to the Helsinki Declaration.

### Echocardiographic measurements

Echocardiographic examinations were acquired using a Vivid 7 scanner (GE Vingmed Ultrasound, Horten, Norway). All analyses were performed blinded to angiographic results.

#### Strain, strain rate and post-systolic index by automated tissue Doppler imaging

**Fig. 2:**
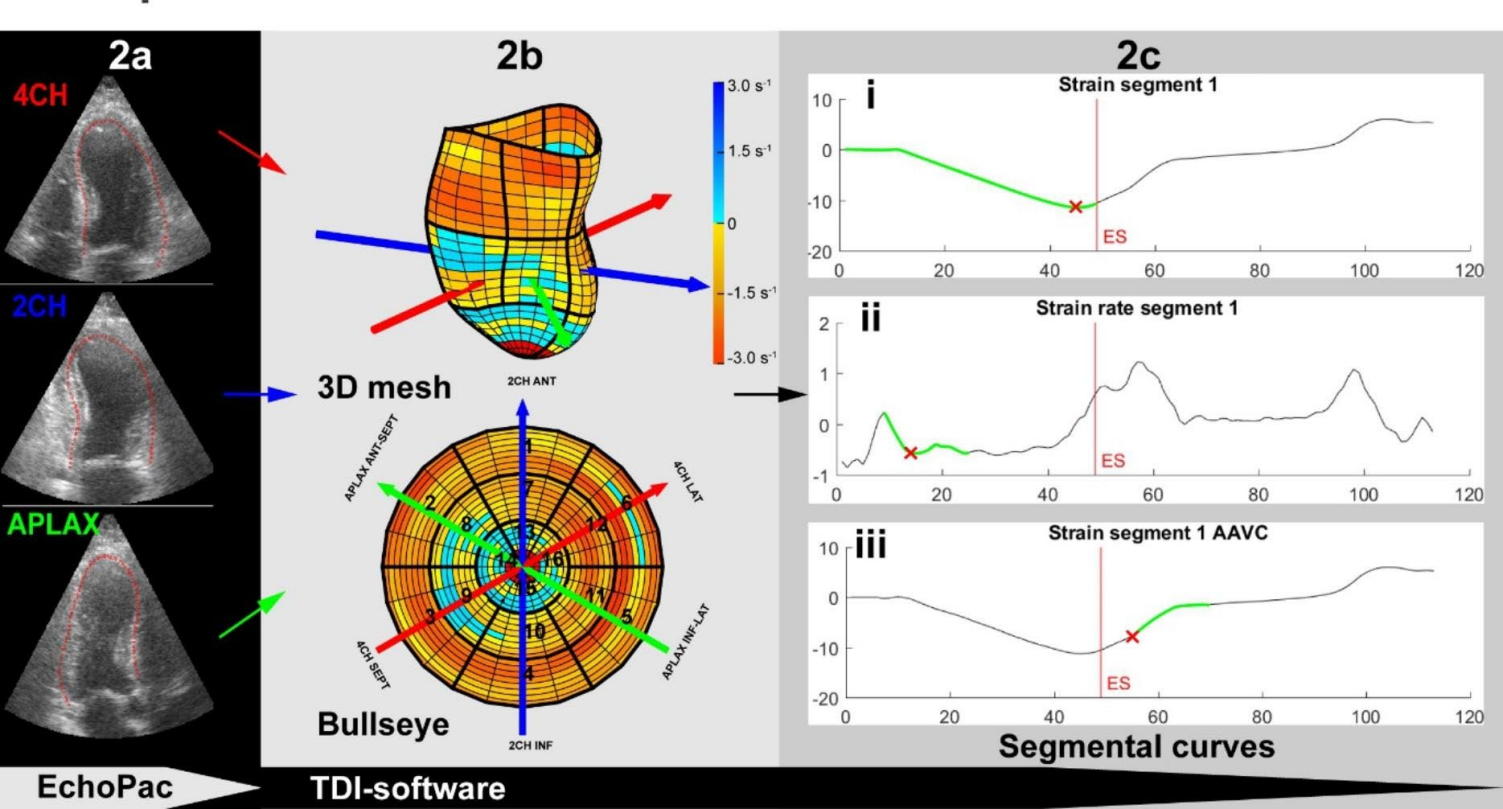
The process from EchoPac to values in the TDI-software. 2a: 3 apical views from EchoPac. 2b: Velocities and position values are extracted by the automated method into a 3D mesh, assuming 60 degrees between imaging planes and a bullseye plot. The colors reflect the strain rate values from red (-3.0 s^− 1^ /contraction) to yellow (0 s^− 1^) to dark blue (3.0 s^− 1^/stretch). Red subsegments show values that are discarded. 2c: Extracted peak values (indicated by red cross) from segmental curves. i: peak systolic strain. ii: peak systolic strain rate. iii: peak post systolic strain. X-axis is frame-numbers, Y-axis is strain (%) for curve i and ii and strain rate(s^− 1^) for iii. The periods in the heart cycle where the peak-values are searched for are marked in green. Red vertical line marks the aorta valve closure defined as the smallest reconstructed volume from the 3D mesh. ES: End Systole. AAVC: After Aorta Valve Closure

To obtain segmental peak systolic and post systolic strain and strain rate values from the TDI software, manual tracing of the left ventricular contour in the tissue Doppler recordings was first performed for each of the three standard apical views (apical four-chamber, two-chamber, and long-axis). A commercially available software package (EchoPac 202x, GE Vingmed Ultrasound, Horten, Norway) was used for the tracing. The traced positions along the myocardium from the three apical views were extracted from EchoPac and imported into the TDI software. The tissue velocity for each position was extracted from the ultrasound DICOM recording. If the myocardial wall and the manual tracing of the greyscale image was outside of the TDI sector, the TDI beam closest to the traced point was considered. Assuming a 60˚ rotation angle between the views, the TDI software applied cubic spine interpolation to calculate the missing data circumferentially between the views to construct a 3D mesh. A standard 16 segment model was applied to the mesh, subsequently per segment velocity and position data was derived automatically, from which global longitudinal strain (GLS), strain rate and PSS were calculated (Fig. 2). The top one-fourth of the apical segments were discarded to avoid reverberations in the apical cap [10]. Details of the TDI software have been published previously [8], however the TDI extraction was implemented as a new plug-in for this project. The TDI software was extended to extract peak segmental values at given timepoints through the heart cycle. For peak systolic strain, the TDI software searched through the entire systole. For peak systolic strain rate, the search range was limited to between 8% and 50% of the systolic length to avoid the pre-ejection spike. The search interval for the post systolic strain and strain rate ranged from 90 ms after aorta valve closure (AVC) until mid-diastole. By starting 90 ms after AVC we avoided that the post-ejection spike was misinterpreted as physiologic PSS [11]. Post-systolic index (PSI) was calculated as: *([peak cycle strain – end systolic strain] / [peak cycle strain])*. Finally, the TDI software estimated the area fraction (%) of the mycoardium with noise values in the TDI signal. Cut-off values for noise were a strain value below − 25% or above 5%.

#### Strain, strain rate and post-systolic index by manual tissue Doppler imaging

**Fig. 3:**
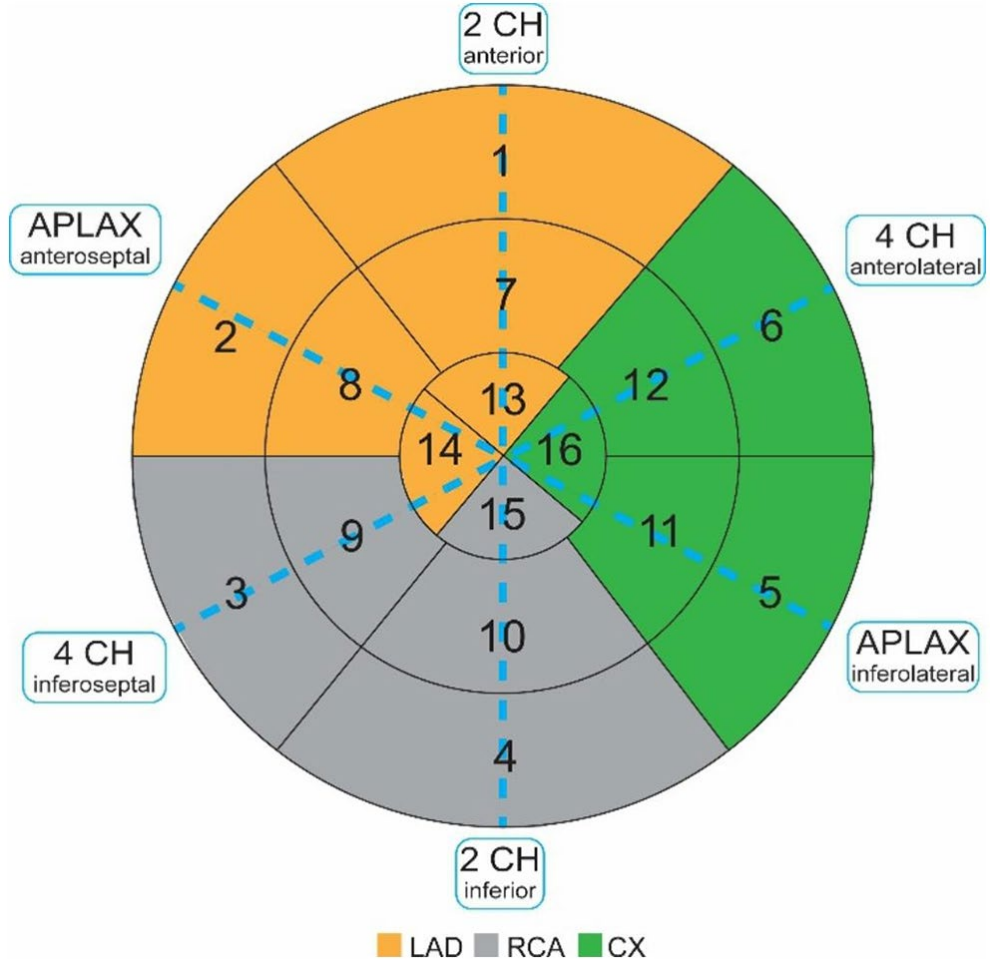
16-segment model adapted from American Heart Association (AHA) [12]. Values from segment 1, 2, 7, 8, 13 and 14 were averaged to Left Anterior Descending artery (LAD) territory, segment 5, 6, 11, 12 and 16 to Circumflex artery (CX) territory and segment 3, 4, 9, 10 and 15 to the Right Coronary Artery (RCA) territory

Manual measurements of TDI-based peak systolic and post systolic strain and strain rate were performed by an experienced reader using EchoPac 202x (GE Vingmed Ultrasound, Horten, Norway). The ROI was placed to best fit the myocardial wall in concordance to the six segments in each of the three apical standard views (Fig. 3). Peak systolic and post systolic strain was manually read from the strain curves corresponding to each segment. As in the automated TDI software, the post systolic peak was considered 90ms after AVC. Peak systolic strain rate values were manually read from the curves in the same heart cycle by using the same ROIs. The AVC timing was determined by Doppler recordings from the left ventricular outflow tract. Segments and curves obviously affected by noise, was rejected.

#### Strain and post-systolic shortening by speckle tracking

Segmental strain and post-systolic index (PSI) were analyzed by a commercially available speckle tracking software (EchoPac 202x, GE Vingmed Ultrasound, Horten, Norway) using the semi-automatic Automated Functional Imaging (AFI) mode. The speckle tracking echocardiography AFI (STE-AFI) software used the three standard apical views and placed the region of interest (ROI) automatically to fit the myocardial wall. The ROI was manually adjusted to best fit the myocardial wall from endocardium to the epicardial border. Special care was taken to not include the pericardium. The tracking quality was visually assessed by comparing the underlying grey scale cine loop with the tracking and the segment was rejected if tracking quality was poor. In a 16-segment model [10, 12], STE-AFI provided mid wall peak systolic strain per segment and segmental PSI. Strain analyses were performed according to recent recommendation papers from EACVI/ASE [13].

#### Territorial values

To calculate territorial strain and strain rate values corresponding to the theoretical perfusion area of the main epicardial coronary arteries, both for TDI and speckle tracking, we averaged segmental values corresponding to each territory [12]. A 16-segments left ventricular (LV) model was constructed (Fig. 3).

### Coronary angiography

Coronary occlusion was defined as TIMI flow 0 or 1 in the infarct-related artery. Angiographic findings were matched with corresponding territorial deformation in the 16-segments model.

### Statistical analyses

Statistical analyses were performed by STATA/MP 17. Descriptive statistics are presented as n (%) for the categorical variables and mean (SD) or median (IQR) for continuous variables. Continuous territorial variables in the culprit area were compared to the non-culprit area with two-sample t-test using groups. The global measurements were compared by linear mixed model with subject specific random intercept and group specific residual variance. We also did receiver operating characteristic (ROC) analyses to evaluate the diagnostic ability of echocardiographic variables.

## Results

Of the 100 patients, 11 (11%) examinations could not be analyzed by STE-AFI due to significant heart rate variation between the three views (n = 10) and geometry changes in cine-loop (n = 1). Five (5%) examinations could not be analyzed by the automated TDI software because the tracing in EchoPac was not feasible due to greyscale and TDI incompatibility (n = 2), geometry changes in the cine-loop (n = 1) and technical issues with the DICOM image files (n = 2). The final data set thus consists of 84 examinations (Fig. 1). Of these 84 patients, 53 (63%) were diagnosed with NSTEMI, 11 (13%) had unstable angina and 20 (24%) had non-coronary chest pain. Seventeen patients (20%) had an occluded culprit artery. Echocardiographic examinations were done one hour (IQR 0.5–3.5) and angiography 29 (± 19) hours after admittance. A summary of the patient characteristics is given in Table 1.

**Table 1:** Patient characteristics

**Baseline characteristics**	
Female gender, *n (%)*	23 (27%)
Diabetes, *n (%)*	9 (11%)
Hypertension, *n (%)*	35 (42%)
Family history, *n (%)*	50 (60%)
Hypercholesterolemia, *n (%)*	33 (39%)
Valvular disease, *n (%)*	2 (2%)
Current smoker, *n (%)*	31 (37%)
Non coronary chest pain, *n (%)*	20 (24%)
Age (years), *mean (SD)*	61 (14)
Max Troponin T pre angio (ng/L), *median (IQR)*	111 (0–635)
Time from admittance to coronary angiography (hours), *mean (SD)*	29 (19)
Time from admittance to echocardiography (hours), *median (IQR)*	1 (0.5–3.5)
**Diagnosis**	
NSTEMI, *n (%)*	53 (63%)
Unstable angina, *n (%)*	11 (13%)
Non-coronary chest pain, *n (%)*	20 (24%)
**Culprit lesion**	
Occluded LAD, *n (%)*	5 (6%)
Occluded RCA, *n (%)*	6 (7%)*
Occluded CX, *n (%)*	7 (8%)*

LAD = Left Anterior Descending Artery. RCA = Right Coronary Artery. CX = Circumflex artery. *One patient had concomitant occlusion of RCA and CX, both considered to be possible culprit lesions

### Echocardiographic measurements

Both GLS and global longitudinal strain rate had lower absolute values when measured by the automated TDI than when measured by manual TDI and STE-AFI. In contrast, there were no significant differences between manual TDI and the STE-AFI echocardiographic measurements. Mean GLS was − 16.7 ± 3.2%, -17.3 ± 3.1%, and − 10.5 ± 1.9%) for measurements by STE-AFI, manual TDI and the automated TDI, respectively.

The absolute territorial strain values in the culprit territories for patients with NSTEMI were lower for the automated TDI measurements compared to the STE-AFI measurements (-10.3 ± 1.7% vs. -15.0 ± 3.7%, p < 0.001) and compared to the manual TDI (-10.3 ± 1.7% vs. -16.5 ± 3.3%, p < 0.001) (Table 2). PSI values in the culprit territories were higher for the automated TDI measurements versus the STE-AFI measurements (19.8 ± 12.7 vs. 8.1 ± 6.2, p < 0.001) as well as the manual TDI. With STE-AFI, GLS and global PSI were significantly impaired in patients with occluded compared to non-occluded culprit lesions (Table 2).

**Table 2:** Global echocardiographic measurements according to occluded or open culprit lesion

	Open *n = 67* *mean (SD)*	Occluded *n = 17* *mean (SD)*	p- value
**Automated tissue Doppler imaging (automated TDI)**			
GLS (%)	-10.6 (1.9)	-10.3 (1.7)	0.57
Global systolic strain rate (1/s)	-0.7 (0.1)	-0.7 (0.1)	0.44
Global PSI (%)	14.6 (6.8)	19.8 (12.7)	0.02*
**Manual tissue Doppler imaging (manual TDI)**			
GLS (%)	-17.5 (3.0)	-16.5 (3.3)	0.25
Global systolic strain rate (1/s)	-1.2 (0.3)	-1.2 (0.2)	0.40
Global PSI (%)	3.7 (4.3)	6.3 (5.6)	0.04*
**Speckle tracking echocardiography Automated Functional Imaging (STE-AFI)**			
GLS (%)	-17.1 (2.9)	-15.0 (3.7)	0.01*
Global PSI (%)	5.1 (4.4)	8.1 (6.2)	0.03*

*p < 0.05. GLS = Global Longitudinal Strain. TDI = Tissue Doppler Imaging. PSI = post systolic index. PSS = post systolic shortening.

Similarly, for STE-AFI, territorial strain in the culprit territory was significantly impaired in patients with occluded compared to non-occluded culprit lesions for all territories (LAD − 14.1 ± 5.6 vs. -18.1 ± 4.2, RCA − 10.4 ± 5.4 vs. -16.5 ± 3.3, CX -11.0 ± 6.4 vs. -16.1 ± 4.3, all p-values < 0.05), the same applies to PSI. In contrast, neither the automated TDI software nor manual TDI measurements were able to identify differences in GLS, global longitudinal strain rate or PSI between patients with occluded and non-occluded culprit lesions (Table 3).

**Table 3:** Comparison of deformation variables between occluded and not occluded arteries in the culprit territory and non-culprit territory. The p-values refers to the differences between occluded and open culprit territory

Culprit Artery	Culprit territory open *mean (SD)*	Culprit territory occluded *mean (SD)*	p-value
**Automated tissue Doppler imaging (automated TDI)**			
Territorial systolic strain (%)			
LAD	-9.4 (2.2)	-8.5 (3)	0.39
RCA	-11.2 (2)	-11.3 (2.2)	0.88
CX	-11.9 (2.5)	-12.3 (3.3)	0.63
Territorial systolic strain rate (1/s)			
LAD	-0.7 (0.2)	-0.6 (0.2)	0.46
RCA	-0.8 (0.2)	-0.7 (0.1)	0.31
CX	-0.8 (0.2)	-0.9 (0.3)	0.67
Territorial PSI (%)			
LAD	14.8 (10.7)	24.5 (15.8)	0.06
RCA	15.6 (8.7)	17.5 (15.3)	0.63
CX	16.6 (11.1)	10.4 (4.1)	0.15
**Manual tissue Doppler imaging (manual TDI)**			
Territorial systolic strain (%)			
LAD	-16.1 (3.4)	-15.5 (3.8)	0.73
RCA	-18.2 (3.7)	-15.7 (2.7)	0.12
CX	-15.7 (5.9)	-17.0 (5.8)	0.60
Territorial systolic strain rate (1/s)			
LAD	-1.1 (0.3)	-1.0 (0.2)	0.27
RCA	-1.3 (0.3)	-1.1 (0.2)	0.14
CX	-1.1 (0.3)	-1.1 (0.3)	0.94
Territorial PSI (%)			
LAD	5.1 (7.0)	10.6 (17.7)	0.13
RCA	3.7 (4.6)	6.0 (9.2)	0.27
CX	4.2 (6.7)	7.2 (9.3)	0.27
**Speckle tracking echocardiography Automated Functional Imaging (STE-AFI)**			
Territorial systolic strain (%)			
LAD	-18.1 (4.2)	-14.1 (5.6)	0.05*
RCA	-16.5 (3.3)	-10.4 (5.4)	0.00**
CX	-16.1 (4.3)	-11.0 (6.4)	0.01*
Territorial PSI (%)			
LAD	6.1 (8.4)	14.9 (18.9)	0.04*
RCA	5 (5.1)	11.8 (9.6)	0.00*
CX	5.6 (6.5)	11.0 (9.1)	0.05*

*p < 0,05 **p ≤ 0,001. PSI = post systolic index. PSS = post systolic shortening

**Fig. 4:**
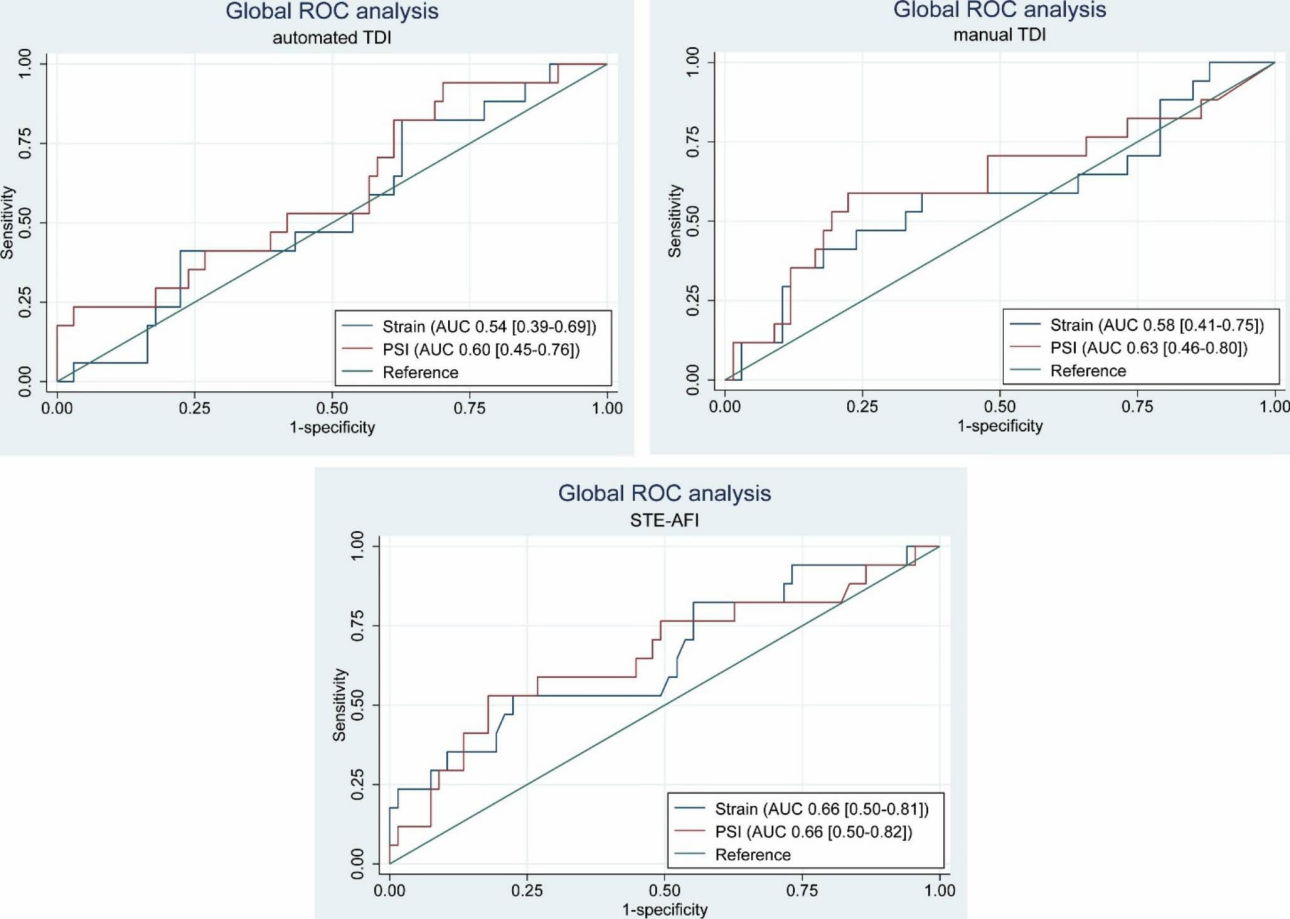
ROC analysis for identification of acute coronary occlusion by global deformation measurements by TDI and STE-AFI AUC [95% Confidence Interval] for the deformation measurements. PSI = Post systolic index

The ROC analysis for GLS showed the same tendency that STE-AFI performed better than TDI. The AUC for TDI is low and suggests that the method might not be useful in a clinical setting (Fig. 4). With STE-AFI, there was a tendency for higher AUC with territorial values compared to global values (Fig. 4; Table 4).

**Table 4:** ROC analysis for identification of acute coronary occlusion by territorial values of strain and post systolic index by tissue Doppler and speckle tracking

	Strain	Strain rate	PSI
	*AUC (95% CI)*	*AUC (95% CI)*	*AUC (95% CI)*
**3Automated tissue Doppler imaging (automated TDI)**			
LAD	0.58 (0.24–0.92)	0.60 (0.30–0.91)	0.74 (0.54–0.94)
RCA	0.38 (0.14–0.62)	0.52 (0.31–0.73)	0.49 (0.26–0.71)
CX	0.44 (0.15–0.73)	0.45 (0.20–0.71)	0.35 (0.20–0.50)
**Manual tissue Doppler imaging (manual TDI)**			
LAD	0.47 (0.19–0.75)	0.65 (0.42–0.87)	0.49 (0.12–0.86)
RCA	0.70 (0.50–0.90)	0.73 (0.58–0.88)	0.48 (0.18–0.77)
CX	0.47 (0.19–0.75)	0.47 (0.2–0.74)	0.55 (0.27–0.83)
**Speckle tracking echocardiography Automated Functional Imaging (STE-AFI)**			
LAD	0.73 (0.51–0.94)		0.73 (0.53–0.92)
RCA	0.85 (0.62-1.00)		0.77 (0.50-1.00)
CX	0.76 (0.51-1.00)		0.68 (0.42–0.94)

PSI = post systolic index.

### Noise

The automated TDI software accepted all segments regardless of tracking and image quality. The number of segments with strain values most likely consistent with noise, ≥ 5% and ˂-25% was 25 (2%) and 20 (1%) respectively. Ten randomly selected examinations had an area fraction average of 4% noise. In contrast, with AFI and the manual TDI method, 378 (28%) and 339 (25%) of the segments were excluded due to poor tracking, noise or out of view motion.

## Discussion

In this study, the inhouse developed TDI software for automated analysis of strain, PSI and strain rate, was not able to identify acute coronary occlusion in patients with suspected NSTEMI. In contrast, strain by STE-AFI accurately identified coronary occlusion and differentiated the occluded territory from the non-occluded territory, in line with earlier studies [1–6, 14], thereby confirming the potential of deformation imaging by STE in early risk stratification of patients with suspected NSTEMI.

### Tissue Doppler vs. speckle tracking

**Fig. 5:**
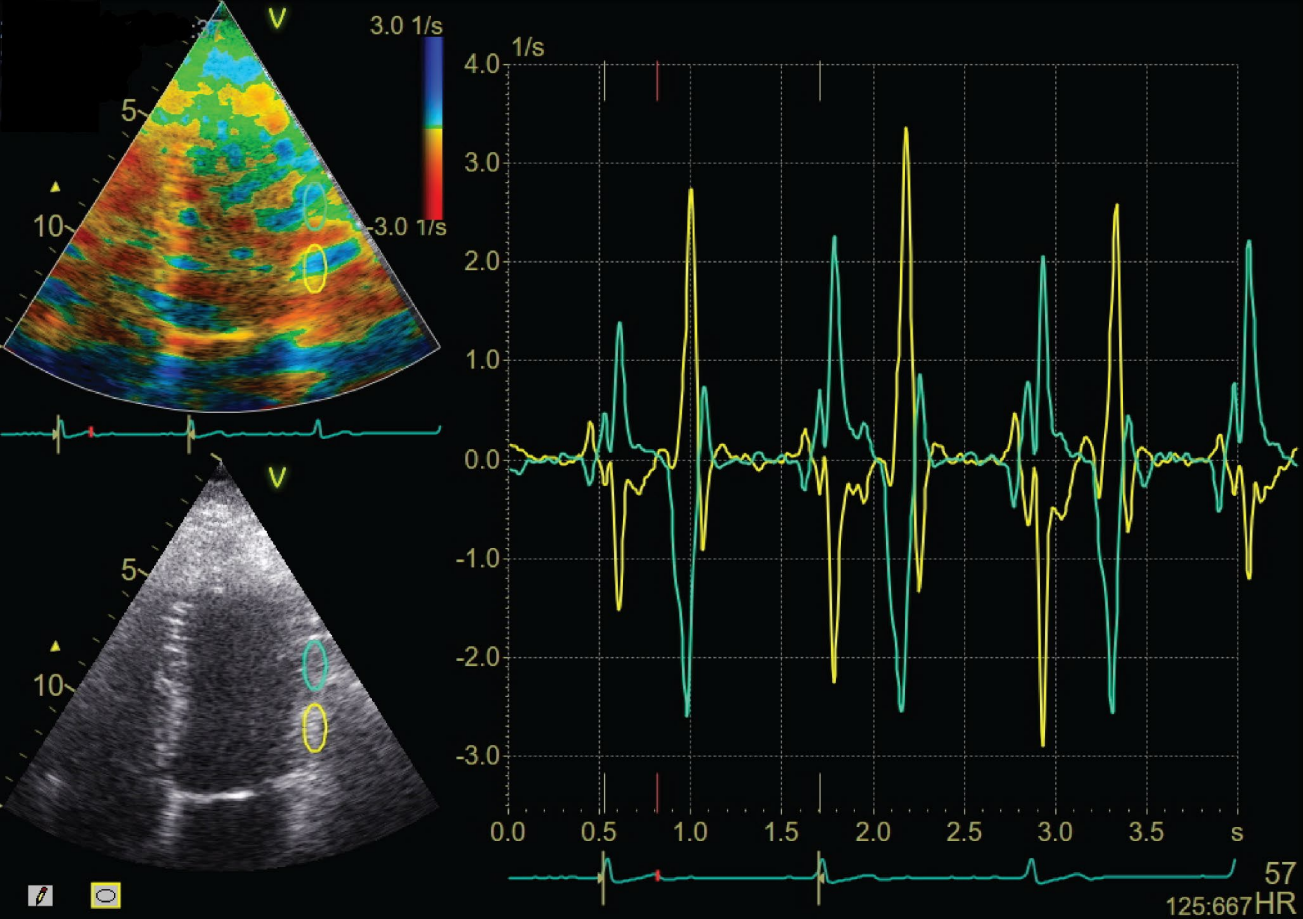
Illustration of effect of reverberations on strain rate curves. The yellow curve is from outside the reverberation whilst the green curve is from inside the reverberation. The size of the region of interest (ROI) is the same. The curve is inverted in the area of the reverberation

The automated TDI software provided lower absolute values than both manual TDI-based measurements and the STE-AFI. This is in line with a comparable study with semi-automatic TDI measurements [15]. Another study revealed that the values were dependent of the territory [16]. An important explanation for discrepant measurements by the automated TDI in the current study might be that the automated software accepts all segments regardless of poor image quality, poor tracking or out of plane motion. In comparison, with the STE-AFI algorithm and the manual TDI, more than one fourth of the segments were rejected. This is comparable to other studies that rejected, dependent of the vendor, 7–23% of the segments [17]. Another explanation is reverberations in TDI images. The automated TDI algorithm chose peak values automatically and in the presence of reverberations, the noise signal and the actual tissue signal can cancel each other out and give values of zero or positive instead of negative (Fig. 5).

Additionally, due to interpolation from the 2D to the 3D model, missing values including noise and reverberations will be extended into the interpolated area. Furthermore, the automated software always analyzed the second of three consecutive cycles, whereas manual measurements allows the operator to choose the heart cycle with best image quality. Together, these factors probably explain why the TDI measurements had significant lower absolute values than the STE and manual TDI.

The image acquisitions consist of numerous frames from each heart cycle. The automated TDI software did averaging of strain values over 5 frames to get more reliable values (current frame and 2 frames back and 2 frames forward as a light form for smoothing). STE smoothing settings are vendor specific and not publicly available. Consequently, we do not know if the smoothing settings are comparable. Smoothing settings will affect the strain measurements [18]. As the manual TDI and STE-AFI not are significantly different, it is plausible to hypothesize that it is the automated software that performs inadequate due to the above-mentioned issues, and not TDI by itself.

### Global versus territorial measurements

Earlier studies have provided conflicting results regarding the diagnostic accuracy of global vs. territorial measurements [19–21]. In this study, territorial deformation measurements had a tendency of better diagnostic ability of coronary occlusion than the global measurements. One reason may be compensatory hyperdynamic contractions in the non-culprit territories in the acute phase of myocardial infarction. Thus, despite regionally compromised deformation, the global deformation is less disturbed. However, this study is too small to further compare global vs. territorial measurement and should only be considered hypothesis generating.

### Clinical implications

This study confirms that strain by speckle tracking echocardiography is a useful tool for identification of coronary occlusion in patients with chest pain. Although TDI has theoretical advantages compared to speckle tracking for strain analyses, further research and technical improvements are still needed to overcome the trade-offs between frame rate vs. image quality, and time-consuming measurements vs. clinical feasibility. In future versions of automated TDI software, implementation of an algorithm for automatic rejection of segments with poor tracking or significant reverberations is paramount.

### Limitations

TDI is susceptible to noise and reverberations. In contrast to STE-AFI, the automated TDI software did not allow for rejection of segments with poor image or tracking quality. Furthermore, the images were acquired on an older generation ultrasound scanner. Modern scanners are available with the possibility of both better image quality and higher frame rates. It is unclear whether new scanners would affect the results in this study. Also, this material constitutes conventional rest echocardiograms only. Future studies must therefore address how these methods perform when assessing the importance of coronary stenosis during stress echocardiography. Finally, the study sample was relatively small with only seventeen patients with occluded arteries. Despite this, the differences between the automated TDI method and STE-AFI seem to be consistent, and at present, speckle tracking remains the method of choice for automated measurements.

## Conclusion

Fully automated measurements of myocardial deformation based on tissue Doppler imaging were not able to identify acute coronary occlusion in patients with suspected NSTEMI. In contrast, semi-automatic measurements of strain by speckle tracking accurately identified coronary occlusion and differentiated the occluded territory from the non-occluded territory. This confirms the potential of deformation imaging in the early risk stratification of patients with chest pain, however improved algorithms are still needed to allow for fully automatic measurements.
